# Influencing Factors of Continuous Use of Web-Based Diagnosis and Treatment by Patients With Diabetes: Model Development and Data Analysis

**DOI:** 10.2196/18737

**Published:** 2020-09-28

**Authors:** Chunhua Ju, Shuangzhu Zhang

**Affiliations:** 1 Business Administration College Zhejiang Gongshang University Zhejiang Province China

**Keywords:** online health communities, patient-doctor trust, ELM, trust theory, structural equation modeling, online continuous diagnosis and treatment

## Abstract

**Background:**

The internet has become a major source of health care information for patients and has enabled them to obtain continuous diagnosis and treatment services. However, the quality of web-based health care information is mixed, which raises concerns about the credibility of physician advice obtained on the internet and markedly affects patients’ choices and decision-making behavior with regard to web-based diagnosis and treatment. Therefore, it is important to identify the influencing factors of continuous use of web-based diagnosis and treatment from the perspective of trust.

**Objective:**

The objective of our study was to investigate the influencing factors of patients’ continuous use of web-based diagnosis and treatment based on the elaboration likelihood model and on trust theory in the face of a decline in physiological conditions and the lack of convenient long-term professional guidance.

**Methods:**

Data on patients with diabetes in China who used an online health community twice or more from January 2018 to June 2019 were collected by developing a web crawler. A total of 2437 valid data records were obtained and then analyzed using correlation factor analysis and regression analysis to validate our research model and hypotheses.

**Results:**

The timely response rate (under the central route), the reference group (under the peripheral route), and the number of thank-you letters and patients’ ratings that measure physicians’ electronic word of mouth are all positively related with the continuous use of web-based diagnosis and treatment by patients with diabetes. Moreover, the physician’s professional title and hospital’s ranking level had weak effects on the continuous use of web-based diagnosis and treatment by patients with diabetes, and the effect size of the physician’s professional title was greater than that of the hospital’s ranking level.

**Conclusions:**

From the patient's perspective, among all indicators that measure physicians’ service quality, the effect size of a timely response rate is much greater than those of effect satisfaction and attitude satisfaction; thus, the former plays an essential role in influencing the patients’ behavior of continuous use of web-based diagnosis and treatment services. In addition, the effect size of electronic word of mouth was greater than that of the physician’s offline reputation. Physicians who provide web-based services should seek clues to patients’ needs and preferences for receiving health information during web-based physician-patient interactions and make full use of their professionalism and service reliability to communicate effectively with patients. Furthermore, the platform should improve its electronic word of mouth mechanism to realize its full potential in trust transmission and motivation, ultimately promoting the patient’s information-sharing behavior and continuous use of web-based diagnosis and treatment.

## Introduction

### Background

In recent years, health care in China has been confronting problems such as increasing numbers of patients with chronic diseases and intensified population aging. With the continuous increase of the number of “netizens” as well as the popularization of web-based health care services [1], the internet has become an essential channel for the dissemination of health care information, providing an opportunity to alleviate the abovementioned problems. Specifically, integration of the internet and health care services has established new communication channels for people to seek medical services on the internet, including health information searches, web-based inquiries, and web-based registration. By assembling high-quality medical resources from various places, the rich information flow allows precise matching between suitable medical service suppliers and patients in need, consequently aiding the balance of medical resource distribution to a certain extent [2]. Furthermore, through web-based inquiry, health counseling, health care activities, and health-themed education, these Web 2.0–based online health communities also provide information support and social support to patients [3,4], exerting positive impact on their self-management of their health and daily disease control through communication [5].

At present, prevention and treatment of diabetes is a major public health problem in China. Diabetes is a chronic and noncommunicable disease that is characterized by long duration, high disability rates, and a wide range of complications [6]. The treatment and control of diabetes is complicated, and long-term professional scientific management is necessary to achieve the goal of reducing acute and chronic complications [7]. Even in daily life, patients with diabetes may experience physical discomfort; obtaining health-related information through the internet can not only cater to patients’ individual needs but can also help decrease costs and greatly increase the efficiency of informational retrieval [8]. By definition, online health communities are places where users can engage in activities such as knowledge sharing and member exchange with regard to health- or treatment-related issues [9]. Web-based inquiry is a vital driving force for the development of online health communities. Physician-patient inquiry is not only the main service provided by online health communities; it is also the most important web-based activity for patients. With guidance in treatment management, medication management, dieting management, etc., communication between patients, family members of patients, and attending physicians is becoming more efficient; this can help patients achieve self-management of their health. The literature on online health communities mainly focuses on knowledge sharing, web-based health information–searching behavior, physician-patient interactions, and web-based health management [10-13]. Few studies have investigated trust and continuous diagnosis and treatment in the context of web-based medical environments. Although there are exceptions, trust is often examined as one of many influencing factors; however, in-depth research of trust is lacking. For example, through questionnaire research, Deng et al [14] found that the credibility of websites, hospitals, and physicians, as well as perceived benefits and perceived risks, have significant effects on the trust of patients who use web-based services. Yi et al [15] designed experiments to examine health information searching behavior and found that the quality of evidence, expertise of the source, perceived information quality, and perceived risk significantly affected users’ trust in network health information. Additionally, web-based inquiry breaks the patient’s constraints in seeking medical care in the sense that that online health communities not only bring together physicians and patients from all over the country to realize mutual assistance regarding knowledge and emotion [16] but also help avoid the embarrassment of face-to-face communication about certain diseases [17].

Prior studies showed that 26% of adult internet users have browsed and viewed others’ published experiences concerning health and medical care [18], and 16% of internet users are willing to find groups with the same health problems through the internet; among these, patients with chronic diabetes are even more inclined to search for disease information and interact with each other on the internet [16]. Therefore, from the perspective of the patient, by analyzing posting data on disease-related questions by patients with diabetes and the top-rated answers from physicians crawled from an online health community and surveyed with a questionnaire, in this paper, we develop a conceptual model to explore factors that influence the continuous use of web-based diagnosis and treatment by patients with diabetes based on the elaboration likelihood model (ELM) and trust theory. The patients’ continuous use of web-based diagnosis and treatment in the present study refers to the patients’ repeated behavior of using an online health community for inquiry, consultation, and help services. Overall, this study not only enriches the strand of empirical research on the web-based interactions, web-based inquiries, and health self-management of patients with diabetes but also has practical implications for the operation and development of online health communities.

### Prior Literature

#### Literature on User Behavior in Online Health Communities

At present, the Chinese literature on user behavior in online health communities mainly comprises various types of behavior, such as information disclosure, information acquisition and searching, information sharing, information service usage and continuous usage, and social support behavior. With regard to the methods, questionnaire surveys and user interviews [19] are the most important approaches for studying user behavior in online health communities in China. However, considering that questionnaire data is subjective in nature and limited in quantity, objective data from the network of the online health community are used in research on user behaviors in these communities. For instance, the impact of user competition on health status was explored in the context of an online weight-loss community [20]. In another study, web-based data from the Good Doctor online health community was obtained using a web crawler, and multiple regression analysis was then conducted to examine the impact of physicians’ and patients’ behavior in online health communities on the knowledge exchange effect [21]. In addition, techniques such as text mining and content analysis have been applied to the analysis of network data, thus expanding the research methods used to study user behavior in online health communities. For example, by applying text mining, users’ question data can be captured based on the keyword *hypertension* and then analyzed; it was found that the informational needs of users in a hypertension health community were mainly concentrated on daily disease management, disease diagnosis and treatment, and the expectation of patients to receive emotional support from society [22]. Applying content analysis to subdivide group behavior into six types for a cancer-themed group on the QQ social media platform, the analysis of different types of behavior showed that the most important types of behavior were emotional support, knowledge sharing, and off-topic behavior [23].

In recent years, the application of social network analysis to research user behavior in online health communities has developed gradually. For instance, based on posting and replying data for half a year as well as on users’ personal information in Tianmijiayuan, an online diabetes health community, Liu et al [24] used an exponential random graph model to explore how network structure and node attributions affect the establishment of users’ reply networks. Zhai et al [16] conducted statistical analysis and social network analysis on user data from the Baidu Quitting Smoking Post Bar; they found that the user group was gradually decreasing in size and that the loss rate was also accelerating. In addition, social network analysis was used to study users’ knowledge-sharing behavior [25] and information dissemination and interaction behavior [26] in online health communities.

#### Physician-Patient Interaction and Patients’ Trust

Physicians are among the most important participants in online health communities. Due to the information asymmetry in online health communities, physicians’ personal information, responses to patients’ consultations, and electronic word-of-mouth reputation can effectively help patients distinguish between physicians at different professional levels to make efficient decisions. For example, some studies have found that a physician’s electronic word-of-mouth reputation, efforts, and service price significantly influence the quantity of their medical inquiry, and the relationship between reputation and inquiry quantity is partially mediated by service price [27]. Moreover, for different diseases, the influencing factors of physicians’ contribution behavior exert varying degrees of impact within different time lengths [28]. Concerning physician-patient interactions, existing research has mainly been conducted from the perspectives of knowledge exchange, physician-patient communication, and physician-patient trust. For example, according to knowledge exchange theory, the impact of behaviors of both physicians and patients (amount of knowledge exchanged, trust, cost, benefits, etc.) on the effectiveness of knowledge exchange was empirically verified [29]. The interaction between physicians and patients was studied from the aspects of the physicians’ degree of activity, patients’ visits, and patients’ satisfaction [22]. By integrating trust factors with perceived benefit and perceived risk, the influencing factors of physician-patient trust in online health services were also explored based on the conceptual framework of web-based trust [14].

Trust is considered to be a basic factor in the formation of successful relationships [30]. In particular, recent studies have focused on the relationship between trust toward providers of products and services and customers’ intentions to make web-based purchases [31]. Among various contexts, patients’ trust can be defined as the patients’ belief and expectation that a medical service provider will take actions that are beneficial to them when they lack the capability to supervise physicians [32]. Previous studies found that trust toward members impacts web-based participation behavior, such as seeking and providing information in focus groups [33]. Hospital rules and regulations as well as the physician’s professional skills and service attitudes will have an impact on patient trust. These factors usually exert roles in the context of medical institutions, medical staff, and medical treatment situations [34]. At the same time, patients’ trust will affect their own health [35]. In particular, information obtained from credible sources is often considered to be more useful and is treated as the basis for decision-making [36].

To conclude, it can be found that literature studies on users in online health communities mainly focus on users’ relationship networks, users’ behavior regarding health information, physician-patient interaction, and patients’ trust. Additionally, some studies have addressed the subjects of methods for calculating similarity among virtual health community users [37], emotional expression of users in online health communities [38], a member’s value co-creation model and its influencing factors [39], and the algorithms of sentiment analysis for user reviews on the internet [40].

### Theoretical Basis and Hypotheses

#### Theory of the ELM

The ELM is a social psychology model. It is a theoretical model that was proposed by Petty and Cacioppo to explain users’ attitudes towards persuasive information changes [41]. It is believed that there are two development routes for changes in personal attitudes: the central route and the peripheral route [41]. The difference between the two routes is mainly reflected in the type of energy input or by information processing differences regarding verbosity. The attitude of the central path mainly comes from a careful evaluation of the information available and the possible benefits of adopting this attitude. This path requires individuals to think critically about the arguments contained in a message and to examine the arguments’ relative advantages and relevance. Then, the judgment of the target behavior is formed. Conversely, in the peripheral route, users mainly rely on tips about the target behavior to make a judgment, such as the number of existing users and information technology experts’ approval.

As a model of persuasion, the ELM is widely used in social psychology, management, marketing, and other fields. Bhattacherjee et al [42] explored how information processing processes affect users’ information technology based on detailed likelihood models and examined how long these effects last. Research finds that quality and information credibility affect user intentions. Filieri and McLeay [43] used a detailed likelihood model to explore information quality, information accuracy, value-added information, and information relevance as influencing factors of the central path related to the timeliness of information and the marginal path related to the product level on the acceptance of information related to accommodation and tourism-related products by tourists. Since the ELM was proposed, many studies have used this model to analyze and understand the process and mechanism underlying users’ information processing in various contexts, such as the user’s knowledge adoption intention [44], the user’s attitude and intention to technology acceptance [45], the consumer’s initial letter for mobile banking [46], and the user’s intention to use the information system [47].

##### Influencing Factors of Patients’ Continuous Diagnosis-Treatment Behavior Under the Central Route

According to the theory of the ELM, when individuals have sufficient motivation and capability, they will think carefully about and make judgments on the quality of argument information; finally, they will form their attitude and behavior towards the information accordingly [48]. For a web-based inquiry service, the homepage of the registered physicians can display evaluations of the service quality by treated patients. These evaluations mainly include patients’ rating of and satisfaction with the physician’s web-based service attitude and service outcomes, respectively reflecting their levels of professionalism and medical technology. As a special commodity service, through web-based medical inquiry, patients often expect physicians to respond quickly to help them solve their personal problems in a timely manner. Usually, if the patients’ satisfaction with the web-based inquiry service and the quality of a physician’s web-based service are high, the risk perceived by the patients will be lower, and they will expect to obtain more professional and effective medical advice and better service. Research shows that informational interaction between consumers and sellers can promote consumers’ trust in sellers and their behavioral intentions [49]. Therefore, a timely response rate, as a characteristic of web-based physician-patient interactions, may positively affect a patient’s online treatment behavior. Therefore, based on the above analyses, the following hypotheses are proposed:

H1: Satisfaction with service attitude has a positive effect on patients’ behavior toward continuous use of web-based diagnosis and treatment.

H2: Satisfaction with service outcome has a positive effect on patients’ behavior toward continuous use of web-based diagnosis and treatment.

H3: Physicians’ timely response rate has a positive impact on patients’ behavior toward continuous use of web-based diagnosis and treatment.

H4: The effectiveness of physicians’ advice has a positive effect on patients’ behavior toward continuous use of web-based diagnosis and treatment.

##### Influencing Factors of Patients’ Continuous Use of Web-Based Diagnosis and Treatment Under the Peripheral Route

Trust propensity varies depending upon an individual’s personality, resulting in different degrees of trust in an environment [50]. Therefore, internet users’ trust in a website is influenced by their personal trust propensity traits. Even for users in the same network, their different trust propensities can lead to different degrees of trust [51]. Studies have found that trust propensity will affect a user’s acceptance of new things, their attitudes towards network communities, and the the degrees of interaction among community members in unfamiliar environments [52]. The higher the individual’s trust propensity, the higher their degree of acceptance of new things or information and the greater their tendency to exchange, communicate, and interact with other community members. A reference group refers to individuals or groups that are closely related to an individual's evaluation, pursuits, or behavior [50]. These groups have an impact on users’ behaviors, lifestyle, attitudes, etc. [53] When users face complex products that lack relevant information, they are more inclined to obtain information from reference groups. Hence, based on the above illustration, an individual’s trust propensity and reference group can be classified as influencing factors under the peripheral route, and the following hypotheses are proposed:

H5: An individual patient’s trust propensity has a positive effect on the patient’s behavior toward continuous use of web-based diagnosis and treatment.

H6: The credibility of the reference group has a positive effect on patients’ continuous behavior toward web-based diagnosis and treatment.

#### Trust Theory

Trust theory was originally proposed by Luhmann [54] to verify changes regarding users’ long-term relationships. This theory has since been applied widely in various disciplines. Trust theory states that users have confidence in network providers, and their willingness to rely on them increases during the process of opinion adoption and purchase payment in the context of shopping on the internet. Existing literature has mainly examined factors that affect web-based consumption, such as product reputation, word of mouth, brand, reliability, etc., and we found that consumer trust is related to factors such as the seller’s reputation, word of mouth, and consumer interest–related guarantee mechanisms [55]. Furthermore, product safety and reliability can influence a customer’s purchase intention by affecting customer trust [56].

##### The Impact of a Physician’s Offline Reputation on Patients’ Continuous Use of Web-Based Diagnosis and Treatment

**A**ll registered physicians in online health communities are required to provide real-name authentication and must display rank information in terms of their professional skill (eg, resident or attending physician) and the hospitals where they work (eg, tier 1C). Generally, the hospital’s ranking represents the medical level of the hospital as a whole. The rank of the physician represents their own professionalism. A higher professional title indicates that the physician has rich clinical experience and a higher degree of professionalism. Therefore, in web-based medical inquiry services, patients believe that a physician with a higher rank who is affiliated with a higher-ranked hospital will have better professional performance and higher authority; therefore, they are more likely to choose that physician. As a matter of fact, a physician’s title is indeed a good indicator of their performance and knowledge. This is usually qualified based upon comprehensive evaluations of a physician’s academic background, work experience, and certificate of their professional qualification examination [57]. As a result, a physician’s title can improve patients’ recognition and trust of that physician and can thus promote patients’ behavioral intentions. Based on the above illustration, the following hypotheses are proposed:

H7: The physician’s professional title has a positive effect on patients’ behavior toward continuous use of web-based diagnosis and treatment.

H8: The hospital’s ranking has a positive effect on patients’ behavior toward continuous use of web-based diagnosis and treatment.

##### The Impact of a Physician’s Electronic Word of Mouth on Patients’ Continuous Use of Web-Based Diagnosis and Treatment

An online health community platform can capture various forms of feedback, such as the number of thank-you letters sent by patients and the patients’ rating of the platform. Specifically, after receiving web-based inquiry services from physicians, patients usually give positive feedback such as thank-you letters and satisfaction ratings to express gratitude or recognize the physician’s professionalism. This type of information is publicly shared on the internet and can improve other patients’ understanding towards the physician, reducing the negative impact of asymmetric information between physicians and patients and helping patients with decision-making. Studies have found that on social media websites, electronic word of mouth can affect users’ purchase intentions through trust transfer and then affect their purchase decisions [58,59]. Based on the above illustration, the following hypotheses are proposed:

H9: The number of thank-you letters has a positive effect on patients’ behavior toward continuous use of web-based diagnosis and treatment.

H10: Ratings by other patients have a positive effect on patients’ behavior toward continuous use of web-based diagnosis and treatment.

**Figure 1:**
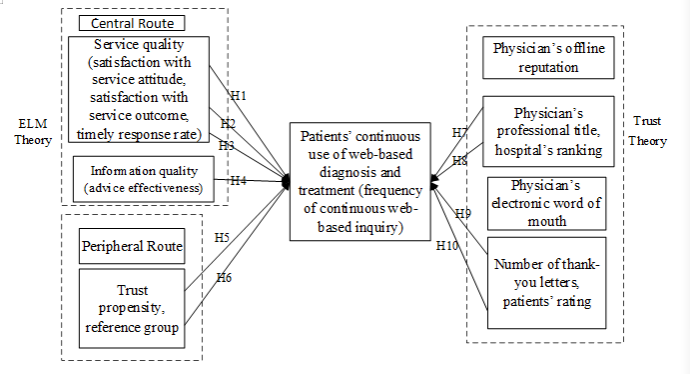
Influencing factor model of behavior toward continuous web-based diagnosis and treatment for patients with diabetes. ELM: elaboration likelihood model.

In summary, public information in a web-based medical community is the main basis for patients’ perception and trust-building towards web-based physicians and determines whether they will choose their services. Therefore, in this study, after controlling for the effect of the platform on the choice of physician by patients with diabetes, we established a conceptual model (Figure 1) in which a patient’s behavior toward continuous use of web-based diagnosis and treatment is conjointly influenced by factors through both the central route and peripheral route as well as by factors such as the physician’s electronic word of mouth and offline reputation. Furthermore, patients with diabetes are considered as a whole in this study, and individual characteristics of the patients are not taken into consideration in this model.

## Methods

### Data Collection

In this study, the data were obtained from an online medical community, WeiYi [60], which archives information about patients’ activities related to web-based diagnosis and treatment. Because patients form their perceptions toward physicians by browsing information on the website and then select a physician for medical inquiry, a web crawler was developed to collect the data. As noted, the focus of the present study is to analyze the influencing factors of the continuous use of web-based diagnosis and treatment by patients with diabetes. The crawler program was written in Python language, and data spanning January 2018 to June 2019 that documented patients with diabetes who had ≥2 medical inquiry records were collected. The data include basic information about physicians and hospitals, the patients’ web-based inquiry procedures, and the patients’ feedback. Furthermore, the patients’ age and sex information were provided by WeiYi authorities. After matching the patients’ ID from the two sources, 4100 pieces of raw data were obtained after excluding missing values. Then, the data information that measure the variables in the conceptual model were identified in a stepwise process to conduct the empirical analysis.

The independent variables were selected based on the conceptual model. The corresponding empirical indicators were selected according to the data provided by the website. The physicians’ offline information (ie, professional title and hospital ranking level) represented the variable of the physicians’ reputation. The indicators for the patients’ central route processing were service quality and information quality, of which service quality was measured by the patient’s satisfaction with the service attitude and service outcome as well as the timely response rate, while information quality was measured by the effectiveness of the physician’s advice. The indicators for the patient’s peripheral route processing were trust propensity and the reference group (ie, feasibility of information provided by the group). The indicators for a physician’s electronic word-of-mouth reputation were the patients’ rating and the number of thank-you letters. The index of service price was obtained directly based on the actual paid price.

The dependent variable is the goal of the research model. As mentioned, the patients’ continuous use of web-based diagnosis and treatment in the present study refers to the patients’ repeated behavior of using the online health community for inquiry, consultation, and help services. Therefore, patients with ≥2 inquiries were selected, and the total number of these patients’ inquiries was measured as the dependent variable to explore its influencing factors.

### Data Analysis

#### Data Preprocessing

Data that met descriptive statistical requirements were obtained through text mining and data cleaning, and data required for correlation analysis and regression analysis were obtained through data transformation. The data preprocessing steps included valid field data matching, character field assignment conversion, null value processing, descriptive statistical analysis, data transformation (normalization and standardization), and outlier rejection.

#### Data Cleaning

According to the variables operationalized in the definition, irrelevant original data were deleted. Then, data collected through the web crawler were matched and integrated with the patients’ ID information. The rules of the character field assignment conversion were as follows. Physicians’ professional titles were ranked as resident physician, attending physician, associate chief physician, and chief physician, corresponding to 1, 2, 3, and 4, respectively. Hospitals were ranked with 9 tiers, and the ranks of the hospitals were classified by searching their full names, in which tier 1C, tier 1B, tier 1A, tier 2C, tier 2B, tier 2A, tier 3C, tier 3B, and tier 3A corresponded to 1, 2, 3, 4, 5, 6, 7, 8, and 9, respectively. The rules of null value processing were as follows. The null value was eliminated if one piece of information regarding the physician’s professional level, hospital ranking level, patient rating score, or price was missing, as this would render the entire piece of information a meaningless outlier. However, if no thank-you letter was found, this was actually of practical significance and was assigned a value of 0, indicating that the physician had not received this type of feedback. In the text of the patient’s review, their opinion of the physician’s professionalism can be described in three ways: extremely professional, very professional, and professional, which were assigned values of 1, 2, and 3. Regarding the trust propensity, the credibility of information provided by patients (the reference group) and the effectiveness of physicians’ advice, a value of 0 or 1 was given, for which 0 indicated low credibility and less effectiveness. After cleaning, 3200 pieces of data were obtained. A sample of the data is shown in Table 1.

**Table 1:** Sample of the data obtained for the study.

Physician’s professional title^a^	Hospital’s ranking level^b^	Thank-you letters, n	Patient’s rating^c^	Satisfaction with service attitude, %	Satisfaction with service outcome, %	Timely response rate, %	Trust propensity^d^	Credibility of reference group^d^	Advice effectiveness^d^	Internet inquiries, n
4	9	20	5	98.8	97.6	67	1	1	1	2
4	9	9	5	100.0	100.0	55	0	1	1	2
3	9	8	5	100.0	78.0	91	1	1	1	2
3	9	0	5	89.0	100.0	100	0	1	1	2
3	9	10	5	100.0	98.6	69	1	0	1	2
2	9	90	4	98.6	100.0	64	1	1	1	3
3	9	40	5	100.0	99.0	100	1	1	0	3
4	9	65	5	97.9	98.8	100	1	1	1	2
4	8	38	5	98.8	99.0	63	1	0	1	4
3	9	40	3	98.0	96.0	66	1	0	0	2
2	9	60	5	99.0	98.8	66	1	0	1	5
2	9	46	4	99.0	98.8	72	0	1	1	2
2	9	97	5	99.0	99.0	78	1	1	1	3

^a^1: resident physician; 2: attending physician; 3: associate chief physician; 4: chief physician.

^b^1: tier 1C; 2: tier 1B; 3: tier 1A; 4: tier 2C; 5: tier 2B; 6: tier 2A; 7: tier 3C; 8: tier 3B; 9: tier 3A.

^c^Patients’ rating: score of 1 to 5, where 1=poor and 5=excellent.

^d^Value of 0 or 1, where 0 is invalid and 1 is valid.

#### Data Transformation

Based on the descriptive statistical results, it was found that the magnitudes of the different variables varied greatly and that the variables showed obviously skewed distribution, excluding the physician’s professional title, hospital’s ranking level, patients’ rating, effectiveness of advice, number of inquiries, and trust propensity and credibility of reference group information. Using square transformation and logarithmic transformation, all data were scaled to the same magnitude, and the skewness was corrected to a certain degree. Within the data boundary, the intervals of different variable indicators intersected. The lower bound of each CI is the difference between the average value and the sampling error, and the upper bound is the sum of the average value and the sampling error. Additionally, outliers that significantly exceeded the range of the variable were removed to reduce noise. Moreover, data that did not fall within the confidence interval were deleted. Finally, 2437 records were obtained for the correlation analysis and regression analysis.

## Results

### Demographic Characteristics

Among the 2437 patients surveyed, 1617 were female (66.4%). This may be because women are often required to care of family health and other responsibilities in addition to work; also, women tend to pay more attention to health information than men [61]. A total of 2060/2437 patients (52.3%) were 20 to 40 years of age. This may be due to specific limitations of the internet use of patients with diabetes. Specifically, diabetes is a chronic noncommunicable disease, and older people may not frequently consult physicians on the internet or ask their family members to perform online inquiries because of their rich personal experiences in consulting physicians and obtaining medicines. In the 2437 records, 1048 of the physicians (43.0%) are chief physicians or associate chief physicians, while 2368 hospitals (97.16%) were ranked 3A. All interviewees had two or more web-based interactions with physicians (see Table 2).

**Table 2:** Summary of the characteristics of the collected data records (N=2437), n (%).

Characteristic		Value
**Gender**		
	Male	820 (33.7)
	Female	1617 (66.4)
**Age** **(years)**		
	20-30	786 (32.3)
	31-40	1274 (52.3)
	41-50	309 (12.7)
	>55	68 (2.8)
**Physician’s professional title**		
	Resident physician	465 (19.1)
	Attending physician	891 (36.6)
	Associate chief physician	848 (34.8)
	Chief physician	200 (8.2)
	Other	30 (1.2)
**Hospital’s ranking level**		
	3A	2368 (97.2)
	Other	69 (2.8)
**Number** **of** **web-based** **consultations** **by the patient** **per year**		
	2	1680 (27.3)
	3	376 (35.1)
	4	205 (14.2)
	>4	176 (4.5)

### Spearman Correlation Analysis

Correlation analysis was conducted on all independent variables to effectively avoid the problem of multiple collinearity of independent variables during multiple regression analysis. Spearman correlation analysis was performed on the final data after cleaning because this method is more suitable for data sets with discontinuous variables. The analysis results showed three pairs of strong correlations. First, a strong correlation was observed between the patients’ rating and the number of thank-you letters, with a correlation coefficient of 0.968. Second, a strong correlation was found between satisfaction with service outcomes and satisfaction with service attitude, with a correlation coefficient of 1; this is consistent with existing research [62]. Last, trust propensity and effectiveness of advice were strongly correlated, with a correlation coefficient of 1. The correlations of all other independent variables were within normal ranges (see Table 3).

**Table 3:** Spearman correlation analysis of all variables (significance level=.05), r.

Variable	Physician title	Hospital ranking level	Timely response rate	Satisfaction with service outcome	Satisfaction with service attitude	Thank-you letters	Patients’ rating	Trust propensity	Reference group	Advice effectiveness
Physician title (*P*<.001)	1	0.040	-0.006	0.012	0.012	0.005	0.004	0.065	0.004	0.016
Hospital ranking level (*P*<.001)	0.040	1	-0.036	0.018	0.018	0.093	0.012	0.063	0.009	0.020
Timely response rate (*P*<.001)	–0.006	–0.036	1	0.089	0.089	0.087	0.056	0.593	0.026	0.023
Satisfaction with service outcome (*P*=.78)	0.012	0.018	0.089	1	1.000	0.548	0.387	0.048	0.017	0.032
Satisfaction with service attitude (*P*=.14)	0.012	0.018	0.089	0.089	1	0.548	0.387	0.163	0.018	0.032
Thank-you letters (*P*=.36)	0.005	0.093	0.087	0.548	0.548	1	0.968	0.029	0.032	0.014
Patients’ rating (*P*=.74)	0.004	0.012	0.056	0.387	0.387	0.968	1	0.065	0.046	0.163
Trust propensity (*P*=.48)	0.065	0.063	0.593	0.048	0.163	0.029	0.065	1	0.034	1.000
Reference group (*P*=.04)	.004	0.009	0.026	0.017	0.018	0.032	0.046	0.034	1	0.008
Advice effectiveness (*P=*.74)	0.016	0.020	0.023	0.032	0.032	0.014	0.163	1.000	0.008	1

### Regression Analysis

Linear regression analysis was performed on all variables, and the results showed that the *R*^2^ value of the model is 0.916. However, when the significance level is .05, the *F* statistics of number of thank-you letters, satisfaction with service outcome, satisfaction with service attitude, patients’ rating, trust propensity, and advice effectiveness are not significant, which indicates that serious multicollinearity is likely to occur. To eliminate collinearity, two groups of control experiments for the three sets of strongly correlated indicators in the correlation analysis results were established. In group A, the indicators of patients’ rating, satisfaction with service attitude, and number of thank-you letters were retained. In group B, the indicators of trust propensity, satisfaction with service outcome, and advice effectiveness were retained. SPSS software (IBM Corp) was used to perform regression analysis on the normalized data set. The results are shown in Table 4.

The experimental results of the two groups were basically the same, indicating that the models were reliable. The *R*^2^ values of both models were greater than 0.68, indicating that the independent variables can account for more than 68% of the change in the dependent variable. Therefore, the regression model had a good fit. The *F* statistic (α=.000, *P*<.005) showed that at the significance level of .05, the linear relationship of the regression model was significant; therefore, the regression model was explanatory for the hypotheses. Based on the model results, the final multiple regression model is as follows:

CQ_A_ = 0.004 * physician’s professional title + 0.018 * hospital’s ranking level + 0.096 * number of thank-you letters + 0.628 * timely response rate + 0.428 * patients’ rating + 0.513 * reference group – 0.798 * satisfaction with service attitude

CQ_B_ = 0.006 * physician’s professional title + 0.019 * hospital’s ranking level + 0.693 * timely response rate + 0.518 * reference group + 0.002 * advice effectiveness – 0.912 * satisfaction with service outcome – 0.013 * trust propensity

**Table 4:** Results of the regression analyses.

Variable	Group A (*R*^2^=0.689; significance level=.000)		Group B (*R*^2^=0.692; significance level=.000)		All variables (*R*^2^=0.916; significance level=.000)	
	Unstandardized coefficient	Significance level	Unstandardized coefficient	Significance level	Unstandardized coefficient	Significance level
Physician’s professional title	0.004	0.000	0.006	0.000	0.472	0.000
Hospital’s ranking level	0.018	0.000	0.019	0.000	0.220	0.000
Number of thank-you letters	0.096	0.000	N/A^a^	N/A	0.001	0.363
Timely response rate	0.628	0.000	0.693	0.000	0.144	0.000
Satisfaction with service outcome	N/A	N/A	-0.912	0.000	0.004	0.783
Satisfaction with service attitude	–0.798	0.000	N/A	N/A	–0.384	0.143
Patients’ rating	0.428	0.000	N/A	N/A	0.000	0.736
Trust propensity	N/A	N/A	–0.013	0.000	–0.043	0.482
Reference group	0.513	0.000	0.518	0.000	–0.019	0.040
Advice effectiveness	N/A	N/A	0.002	0.000	–0.028	0.744

^a^N/A: not applicable.

## Discussion

### Main Findings

In this section, we discuss the hypotheses regarding the influencing factors of patients’ continuous use of web-based diagnosis and treatment based on the above empirical results. Generally speaking, according to the analytical results, the timely response rate, reference group, and patients’ rating are the three indicators with the largest positive regression coefficients, indicating that the timeliness of a physician’s service has a great effect on a patient’s choice and that other patients’ rating scores and information reliability also greatly affect a patient's continuous use of web-based diagnosis and treatment. Furthermore, the coefficient of the hospital ranking level is very small; this may be related to the fact that patients generally choose physicians in hospitals that rank at tier 3a for web-based inquiry.

Hypotheses H1 through H6 can be discussed from the perspective of ELM theory. The physician’s service quality (operationalized as satisfaction with service outcome, satisfaction with service attitude, and timely response rate) is an important factor that affects patients’ continuous use of web-based diagnosis and treatment through the central route. The data show that attitude satisfaction and effect satisfaction have negative effects on patients’ continuous use of web-based diagnosis and treatment, and the regression coefficients are large. The data also show that timely response rate has a significant positive effect on patients’ continuous use of web-based diagnosis and treatment, while advice effectiveness presents a small effect. Therefore, the analytical results deny H1 and H2 and support H3 and H4. These findings can be explained in two ways. On the one hand, patients do not select physicians with high satisfaction ratings incautiously. On the other hand, in contrast with other diseases, diabetes is a disease that requires chronic long-term treatment; therefore, patients with diabetes may be more concerned about the quality of service from the perspective of response timeliness. For H5 and H6, the data show that the reference group and trust propensity have significant effects on patients’ continuous use of web-based diagnosis and treatment through the peripheral route; the effect size of the reference group is greater than that of trust propensity, providing supportive evidence for both hypotheses. These results reveal that when patients lack motivation and capability to judge the information quality provided by physicians in online health communities, or when a clear view is not yet formed, a credible reference group is a very convincing indicator of the patients’ decision. This is consistent with the conclusions in existing literature studies of the effects of reference groups on consumer behavior, lifestyle, self-concept development, and attitude [63].

From the perspective of trust theory, H7 through H10 can be discussed. The statistics show that a physician’s professional title and hospital ranking level are positively related with patients’ continuous use of web-based diagnosis and treatment, supporting H7 and H8. The significance of the relationship suggests that trust in a physician’s offline reputation will influence trust in their web-based services. In other words, trust in offline medical services can be directly transferred to web-based medical services. However, the low coefficients further indicate that patients are less susceptible to a physician’s offline characteristics after receiving multiparty information [62]. The statistics also show that the number of thank-you letters and the patients’ rating are positively related with the number of uses and have a greater impact on patients’ continuous use of web-based diagnosis and treatment, supporting H9 and H10. The significance of relationships suggests that patients care more about physicians’ electronic world of mouth because they believe that it is an indicator that condenses other patients’ internet experiences. The finding that a physician’s electronic word-of-mouth reputation can help patients make decisions is consistent with the research findings of Cao et al [64]. At the same time, this research also reflects that trust has an overall positive effect on the continuous use of web-based diagnosis and treatment by patients with diabetes, which demonstrates that patients’ trust in web-based inquiry services is highly significant in predicting whether they will choose web-based consultation services.

### Theoretical Contributions and Practical Implications

The present study makes several theoretical contributions.

First, few previous studies have comprehensively applied ELM theory and trust theory together to examine web-based inquiry services; also, these studies seldom take price and credibility of the trust source into consideration for analysis. By filling this research gap, our research contributes to new ideas regarding patients’ behavior related to web-based diagnosis and treatment. We found that a physician’s offline reputation has a relatively weak influence on patients’ decision-making with regard to continuous use of web-based diagnosis and treatment. On the one hand, when using the web-based inquiry service, among the physicians and hospitals selected by patients, 55.4% of physicians are chief physicians or associate chief physicians and 98% of hospitals are in the top tier (3a). It can be concluded that the level of professional skill does not significantly influence patients’ decision-making because this type of information is usually available to patients on the internet without distinction. On the other hand, unlike offline interactions with physicians, patients mainly make web-based inquiries for the purpose of obtaining disease-related information. After receiving information from multiple sources, they can make a wise decision as to which physician to choose. Furthermore, the acquisition of information is not restricted by region. Thus, the role of the physician’s offline reputation is no longer significant.

Second, the core factor that influences patients’ continuous use of web-based diagnosis and treatment is the physician’s electronic word-of-mouth reputation. Patients can preliminarily perceive physicians’ service attitudes and professional skill through the timeliness of their responses and their professional titles. Then, combined with other patients’ ratings of physicians’ receptive attitude and service efficacy, patients will establish their own trust toward the physicians, which will aid their subsequent decision-making. This finding suggests that patients place much more value on the timeliness of web-based inquiry. Previous studies have shown that the impact of positive evaluation on purchase intention is less than that of negative evaluation on customers’ refusal to purchase [65], and both the quality and number of evaluations affect users’ purchase intentions [66]. Our data illustrate that the average score of satisfaction with the physicians is quite high (98%), and only a few records show satisfaction below 60%. Taken together, these analyses reflect that the satisfaction scoring mechanism of the platform is not well designed, leading to a decrease in the reliability of the satisfaction score and likely consequently exerting a negative impact on patients’ decision-making.

Building upon these findings, several practical implications can be summarized to improve actual service. First, in terms of physicians who are registered on the internet, to improve patients’ rate of use of their services, it is key for the physicians to ensure the quality of their responses to patients’ web-based inquiries and consultations as well as to increase their activity on the platform. Further, physicians are encouraged to provide complete supplementary information regarding their professional experiences. Second, in terms of web-based platform building, the feedback mechanism should be optimized. This is because patients pay more attention to the number of thank-you letters and ratings by other patients when making decisions. At the same time, the indicators that measure physician-patient interactions, such as timeliness of response, should be much more scientifically refined. The mechanism related to electronic word-of-mouth should be improved to reflect its role in trust transmission and encouragement. For instance, the control of satisfaction scoring should be strengthened to reduce or avoid adverse network behaviors such as click farming, ensuring the quality of web-based inquiry information. Third, in terms of the relationship between platforms, physicians, and patients, compared with the ranking of the hospital, the physician’s professional title has a greater impact on patients’ decision-making. This implies that the platform should strengthen and broaden its cooperation with patient-trusted physicians who offer high-quality services and that specific needs for behavior related to web-based diagnosis and treatment and the underlying psychological mechanisms for patients with different diseases should be further carefully considered.

### Limitations

The present research is not without limitations. First, the data were obtained from a single web-based medical community, WeiYi, which decreases the generalizability of the findings. In future research, collection of data from multiple online health community platforms should be considered to verify the results. Second, this study is focused on factors that influence the behaviors of Chinese patients with diabetes regarding web-based diagnosis and treatment. In the future, similar studies should be carried out in western regions. Third, regarding the research methods, future studies should take various regression methods into consideration and conduct comparative analyses. For instance, in addition to the regression methods used in this study, baseline regression analysis containing all variables can also be applied, and follow-up studies on factors that moderate patients’ behavior regarding web-based services can be conducted by introducing patients’ experiences with web-based inquiries, involvement in social networks, etc.

### Conclusions

In this study, we collaborated with the operators of an online health community by crawling backend log data and obtaining patients’ basic information (age and gender) from the platform; the results of this study help provide a reference for how to optimize web-based inquiry services and enhance patients’ continuous behavior related to diagnosis and treatment. As a necessary means to alleviate the problem of restricted offline medical resources, web-based inquiry services, which are not constrained by time and space, have improved the satisfaction of patients with diabetes regarding their self-management of their health. However, the actual adoption rate of web-based inquiry services by patients with diabetes remains low. Applying ELM theory and trust theory, the results of our study show that the timeliness of physicians’ responses to web-based inquiries is positively related to patients’ continuous use of web-based diagnosis and treatment, leading to high intention of patients to continue to use web-based inquiry services; the reference group is conducive to patients’ trust towards and continuous use of web-based inquiry services; and the effect sizes of hospital ranking levels and physicians’ professional titles are small. Overall, the conceptual model showcases good explanatory power for predicting continuous use of web-based diagnosis and treatment by patients with diabetes, expanding the literature on mobile medical services. The findings provide important implications regarding ways to promote healthy development of online health communities and to help solve the problems of resource shortages and information asymmetry in the medical industry.

## Abbreviations

ELM: elaboration likelihood model
